# Molecular typing of the pneumococcus and its application in epidemiology in sub-Saharan Africa

**DOI:** 10.3389/fcimb.2013.00012

**Published:** 2013-03-14

**Authors:** Eric S. Donkor

**Affiliations:** Department of Microbiology, University of Ghana Medical SchoolAccra, Ghana

**Keywords:** pneumococcus, multilocus sequence typing, sub-Saharan Africa, eBURST, epidemiology

## Abstract

Molecular typing of the pneumococcus has played a crucial role in understanding the epidemiology of the organism. However, most of what is known about molecular epidemiology of the pneumococcus pertains to the developed world. The brunt of pneumococcal infections is borne by sub-Saharan African countries, which makes epidemiological monitoring of the pneumococcus essential in this region of the world. This review paper focuses on molecular typing of the pneumococcus and what is known about epidemiology of the organism in sub-Saharan Africa based on the various typing methods. Several molecular typing methods are available for typing the pneumococcus and the major ones include multilocus sequence typing (MLST), multilocus enzyme electrophoresis (MLEE), serotyping and DNA fingerprinting methods such as pulsed field gel electrophoresis (PFGE) and amplified fragment length polymorphism (AFLP). Currently, MLST is the most suitable method for typing the pneumococcus. The pneumococcal population structure in sub-Saharan Africa appears to be quite different from that of the developed world, and pneumococcal serotype 1 related to the ST 618 clone and clones of the ST 217 clonal complex are responsible for outbreaks in sub-Saharan Africa.

## Introduction

*Streptococcus pneumoniae* is part of the normal bacterial flora of the upper respiratory tract, but is also associated with severe invasive diseases, including meningitis, pneumonia, and septicaemia as well as non-invasive diseases such as otitis media (Mitchell, 2006). An important feature of *S. pneumoniae* is the presence of a polysaccharide capsule which is the basis of 94 serotypes of the organism (Henrichsen, 1995; Park et al., 2007; Jin et al., 2009; Calix and Nahm, 2010). *S. pneumoniae* is a naturally transformable organism, indicating that it can gain DNA from exogenous sources and incorporate it into its genome. This facilitates evolution of *S. pneumoniae* which occurs by horizontal gene transfer and leads to evolutionary phenomena such as capsular switching and antibiotic resistance. Occurrences of these evolutionary phenomena as a result of pneumococcal conjugate vaccinations have been documented (Brueggemann et al., 2007; Pelton et al., 2007).

Molecular typing of bacterial pathogens has become very important in studying the epidemiology of bacterial diseases, and many of such typing schemes have been developed over the years. For the pneumococcus, molecular typing has provided invaluable insights into analyzing the clonal spread during epidemics, identifying infection route and transmission, and assessing evolutionary changes following vaccination (Spratt et al., 2004). However, most of what is known about molecular epidemiology of the pneumococcus emanates from the developed world. Majority of pneumococcal infections occur in sub-Saharan Africa (Black et al., 2010), which makes epidemiological monitoring of the pneumococcus essential in this region of the world. Additionally, massive pneumococcal vaccination is expected in sub-Saharan Africa (PneumoADIP, 2007), and therefore a better understanding of the genetic background of *S. pneumoniae* in the region is required. This review paper summarizes the various molecular methods used in typing the pneumococcus and what is known about epidemiology of the organism in sub-Saharan Africa based on the typing methods.

## Molecular typing methods of *Streptococcus pneumoniae*

### Serotyping

Currently, the main *S. pneumoniae* serotyping methods include the Quellung reaction, latex agglutination, and PCR-based methods. Quellung reaction is considered the gold standard for serotyping of *S. pneumoniae* and other encapsulated bacteria such as *Klebsiella pneumoniae*, *Haemophilus influenzae*, and *Neisseria meningitidis* (Baek et al., 2011). Using the Quellung reaction for serotyping is very reliable and reproducible, but also it is labor-intensive. There are several types of the latex agglutination method used for serotyping of *S. pneumoniae*. However, the latex agglutination test developed in 2004 by the Statens Serum Institute in Denmark (Slotved et al., 2004) is used as a standard method for pneumococcal serotyping in many laboratories. Generally, the latex agglutination serotyping method is simple and fast, but is not suitable for detecting multiple serotype carriage. PCR-based serotyping methods exploit the heterogeneity within the capsular operon of different serotypes (Bentley et al., 2006). The first PCR-based serotyping technique for *S. pneumoniae* was a single multiplex reaction developed by Brito et al. (2003) and could accurately determine six common serotypes and two serogroups (collection of related serotypes). Subsequent multiplex serotyping assays have been expanded to cover more serotypes (Pai et al., 2006; Pimenta et al., 2009), but up to a maximum of 33 can be identified based on the method developed initially by Pai et al. (2006) and modified by the Centre for Disease Control in Atlanta (http://www.cdc.gov/ncidod/biotech/strep/pcr.htm). Generally, PCR-based serotyping has been shown to be reliable and accurate, and can often be used directly on clinical specimen. However, a key limitation of current PCR-based typing methods is the inability to distinguish between many serotypes of the same serogroup (Brito et al., 2003).

Recently, a microarray-based genomic tool for molecular serotyping of *S. pneumoniae* based initially on the complete sequences of the capsular loci of 91 serotypes has been developed (Turner et al., 2011). The capsular microarray gives a high level of serotyping accuracy (95–100%), but is limited in differentiating some closely related serotypes (Turner et al., 2011).

Generally, serotyping has a poor discriminatory ability as it is based on a single locus. Additionally, capsule switching has been observed with the pneumococcus, which means that capsule does not relate well to genetic background. The poor discriminatory ability of serotyping makes it less useful for molecular epidemiological studies. Despite this, serotyping is likely to remain an important typing method for pneumococci as both natural immunity and current pneumococcal vaccines are based on the capsule types of the organism.

Latex agglutination is the serotyping method used in most microbiology laboratories in sub-Saharan Africa, and one sample cost about US$29. Serotyping by the Quellung reaction and PCR cost about US$21 and US$8, respectively.

### DNA fingerprinting methods

These methods compare DNA fragment patterns generated by restriction endonuclease enzymes, or in combination with DNA PCR amplification. The various DNA fingerprinting methods of *S. pneumoniae* include Pulsed Field Gel Electrophoresis (PFGE), Penicillin Binding Protein (PBP) Fingerprinting, BOX-PCR, Restriction Fragment Length Polymorphism (RFLP), and Amplified Fragment Length Polymorphism (AFLP). PFGE, a genotypic method for the evaluation of total chromosomal DNA, had been regarded as the “gold standard” for typing micro-organisms (Schwartz and Cantor, 1984). PFGE has been reported to have similar discriminatory ability as some PCR fingerprinting methods such as BOX-PCR and RFLP (Yu et al., 2003). AFLP has however, been reported to yield better discrimination between pneumococcal and non-pneumococcal species compared to PFGE (Neeleman et al., 2004). PFGE and other DNA fingerprinting methods possess appreciable levels of discrimination and reproducibility, and can be used to address more short term evolutionary questions. However, they are unsuitable for population structure analyses as they index genetic variation whose origin are not properly understood and are under diversifying selective pressure (Spratt, 1999). Additionally, the DNA fingerprinting methods would be challenging to compare between different laboratories.

### Multilocus enzyme electrophoresis

Multilocus enzyme electrophoresis (MLEE) explores the different electrophoretic mobilities of a large number of cellular enzymes encoded by house-keeping genes (Selander et al., 1986). MLEE has a high discriminatory ability and also uses genetic variation that is selectively neutral and accumulates slowly. These features make MLEE highly efficient in population structure analysis of bacteria, and in several studies, the technique has been successfully employed to investigate *S. pneumoniae* (Hall et al., 1996; Combe et al., 2000). The main drawback of MLEE is that it does not determine genotypes but phenotypes, which are susceptible to environmental influences. This can adversely affect the reproducibility of MLEE results, and therefore, the MLEE data sourced from different laboratories tends not be comparable. Additionally, conservation of housekeeping genes makes MLEE unsuitable for population studies of bacteria of a more clonal population structure.

### Multilocus sequence typing

Multilocus sequence typing (MLST) shares the same principle with MLEE but assigns alleles to housekeeping genes directly by sequencing of those genes, and thus the level of resolution is much better than MLEE (Spratt, 1999). For each gene, the distinct sequences present within a bacterial species such as *S. pneumoniae*, are assigned as distinct alleles, which are further used to define an allelic profile or sequence type (ST) of a strain. So far, over 8000 *S. pneumoniae* STs from a wide variety of geographical locations have been described in the *S. pneumoniae* MLST database http://speumoniae.mlst.net/. However, there is little MLST data on African isolates. The major advantage of MLST is that sequence data is unambiguous and the STs of isolates can be compared to those in a central database via the Internet, in contrast to other typing methods which involve comparing DNA fragment sizes on gels. Thus the portability of MLST means it is highly useful in monitoring the population structure of a given pathogen. Due to the sequence conservation in housekeeping genes, sometimes MLST has limited ability to differentiate bacterial strains, which limits its use in the epidemiological investigation of bacteria of a more clonal population structure. For a naturally transformable organism like *S. pneumoniae*, MLST is a valuable typing tool, as the high rates of recombination result in a high rate of genetic diversification of pneumococcal populations (Brueggemann et al., 2004; Hanage et al., 2005). In terms of cost, MLST is relatively more expensive compared to other molecular/genotyping methods such as MLEE and PFGE. It cost about 15 USD to analyze one pneumococcus sample by MLST, and a complete analysis would also involve serotyping cost of US$29 (latex agglutination). The high cost of MLST is likely to be a major limitation to its use in sub-Saharan Africa.

### Molecular epidemiology of *S. pneumoniae*

Over the years, the various molecular typing schemes of pneumococcus have greatly facilitated our understanding of the molecular epidemiology of the organism. Serotyping data indicates that only a few of the discovered 94 serotypes are responsible for the vast majority of pneumococcal disease, and these serotypes may vary from one geographical region to another (Hausdorff et al., 2000; Hausdorff, 2007). A recent global review on pneumococcal serotypes causing invasive diseases in 51 countries showed that serotype 14 was the most common in all regions including Africa, Asia, Europe, Latin America, Northern America, and Oceania (Johnston et al., 2010). In this review, it was observed that seven serotypes (1, 5, 6A, 6B, 14, 19F, and 23F) accounted for 66–76% of diseases in each region, and the top eight serotypes in Africa and Asia were the same which included, the seven serotypes mentioned in addition to serotype 19A. It was also observed that serotype 14 was the dominant serotype in younger children (<23 months), while serotype 1 was the dominant serotype in older children (24–59 months).

Genotyping of pneumococci has provided further insights into the molecular epidemiology of pneumococcal serotypes. One of such insights is that isolates of certain serotypes such as 1 and 7F are associated with a limited number of clones, and these clones are hardly observed with other serotypes (http://spneumoniae.mlst.net/). On the other hand, serotypes such as 9V and 14 are associated with a wide variety of clonal types, which may also be seen with some other serotypes (http://spneumoniae.mlst.net/). The Pneumococcal Epidemiology Network (PMEN) provides surveillance on epidemiologically significant clones and currently, 43 such clones are known (http://www.sph.emory.edu/PMEN/index.html).

In sub-Saharan Africa, most molecular epidemiological studies on *S. pneumoniae* have focused on serotype analysis (Cadoz et al., 1981; Campbell et al., 2004; Traore et al., 2009; Donkor et al., 2010) and the major serotypes reported by these studies are similar to that reported generally for Africa (1, 5, 6A, 6B, 14, 19A, 19F, and 23F) by Johnston et al. (2010). Additionally, some serotypes including 2, 9V, and 12 are also important causes of invasive pneumococcal disease in the sub-region (Cadoz et al., 1981; Campbell et al., 2004; Traore et al., 2009). As serotyping has a poor discriminatory ability unlike MLST, these studies provide little information on molecular epidemiology of *S. pneumoniae* in sub-Saharan Africa. The few studies that have applied genotyping to study *S. pneumoniae* in sub-Saharan Africa seem to be more related to outbreaks situations. In The Gambia, Antonio et al. (2008a) studied the molecular epidemiology of 127 invasive and carriage *S. pneumoniae* isolates from unvaccinated children and children vaccinated with a nine valent pneumococcal vaccine which had serotypes 1, 4, 5, 9V, 14, 18C, 19F, 23F, and 6B). Twenty-seven different serotypes and 72 different STs were identified, and there was no particular association of clones with specific presentation of invasive pneumococcal disease. For a given serotype, the genotype distribution varied considerably between vaccinated and un-vaccinated children. However, the population structure of serotype 1 obtained from vaccinated and non-vaccinated children were the same and comprised mainly ST 618. In The Gambia, an outbreak of pneumococcal pneumonia and septicaemia caused by serotype 1 occurred between 1997 and 2002 (Antonio et al., 2008b). In this outbreak, a 127 serotype 1 isolates were recovered and MLST showed that 72.7% of the isolates were of ST 618. The other common serotype 1 clones identified in this study were ST 3575 (9.4%), ST 217 (3.9%), and ST 612 (2.4%). It was observed that ST 618 which caused both pediatric and adult disease peaked annually in the hot dry season, which was not observed for non-ST 618 clones. Prior to the outbreak reported in The Gambia, ST 618 was implicated in a meningitis outbreak in Burkina Faso over the period 2002–2005 (Yaro et al., 2006). In this outbreak, 249 (44%) of 571 cases of bacterial meningitis were caused by *S. pneumoniae* and the associated case fatality rate was 46%. A total of 48 *S. pneumoniae* isolates were serotyped, and 21 (44%) were found to be serotype 1. MLST was carried out on only three of these serotype 1 isolates, and two were found to be ST 618 with the other isolate being a novel ST, and a single locus variant of ST 618. An outbreak of pneumococcal meningitis similar to the outbreak in Burkina Faso also occurred in Ghana during the period of 2002–2003 (Leimkugel et al., 2005). In this outbreak, the case fatality was 44%, and examination of the *S. pneumoniae* isolates responsible for this outbreak showed that 76% were of serotype 1 and were of three STs including ST 217, and its 2 single-locus variants ST303 and ST612. ST 217 which was the dominant clone in this outbreak is also a double locus variant of ST618 responsible for pneumococcal outbreaks in The Gambia and Burkina Faso. The pneumococcal outbreaks described above underscore the clinical importance of pneumococcal serotype 1 in sub-Saharan Africa and show that the ST 618 clone and clones associated with the ST 217 clonal complex are responsible for outbreaks in the region. Pneumococcal carriage of these clones among healthy people has been reported in several countries in sub-Saharan Africa such as The Gambia (Antonio et al., 2008b) and Nigeria (Adetifa et al., 2012).

**Figure 1:**
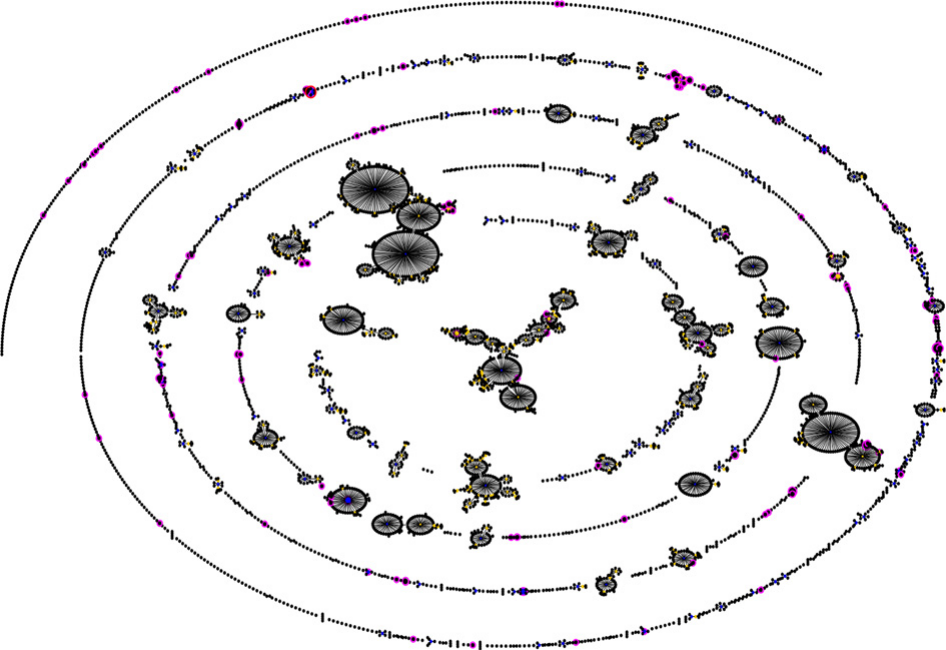
**Comparison of a Gambian pneumococcal population sample with that of the global pneumococcal MLST database.** The figure was generated using eBURST (Feil et al., 2004). STs of the Gambian population are shown in pink, while the blue and yellow colors represent primary founders and subgroup founders, respectively. Majority of the Gambian strains are not associated with the major clonal complexes of the MLST database indicating that the Gambian pneumococcal population sample is thus divergent (Donkor et al., 2011).

Pneumococcal serotype 1 is also an important cause of invasive disease and epidemics in the developed world (Brueggemann and Spratt, 2003). However, while pneumococcal serotype 1 infections in sub-Saharan Africa are more associated with MLST genotypes STs 217 and 618 (Leimkugel et al., 2005; Yaro et al., 2006; Antonio et al., 2008b), in Europe and the United States, STs 306 and 227 are the dominant genotypes causing serotype 1 related infections (Brueggemann and Spratt, 2003). Another example of this situation is with pneumococcal serotype 3 which is an important cause of invasive disease in Europe and the United States, as well as in sub-Saharan Africa; in Europe and the United States, serotype 3 infections are associated with ST 180 (http://speumoniae.mlst.net/), whereas in sub-Saharan Africa, the infections seem to be associated with STs 458 and 700 (Adetifa et al., 2012; Donkor et al., 2013). These observations suggest the possibility of a divergent pneumococcal population in sub-Saharan Africa (compared to pneumococcal population in the developed world) which has been elucidated by a recent pneumococcal carriage study by Donkor et al. (2011) in The Gambia. In this study 148 pneumococcal isolates from children were typed by MLST and 76 STs were identified. The investigators carried out eBURST analysis (a method of finding clonal complexes or relationships between strains by examining MLST profiles) on their isolates in comparison with the pneumococcal MLST global database and observed that the Gambian isolates formed a divergent and diverse population (Figure 1). The investigators also compared the extent of genetic recombination in the Gambian pneumococcal population with those of two European pneumococcal populations from Finland and the UK, and observed that recombination in the Gambian population (34%) was far higher than those in the Finnish sample (2%) and UK sample (5%). It was concluded that the high amounts of recombination in the Gambian pneumococcal population accounts for its divergence compared to pneumococcal samples from other geographical regions. The high amount of recombination among the Gambian isolates is due to horizontal gene transfer which is facilitated by the relatively high rates of multiple pneumococcal carriage in sub-Saharan Africa (Donkor et al., 2011). The high amount of recombination can produce novel combinations of alleles at the MLST loci resulting in the divergent population observed in the Gambian sample. Generally, pneumococcal population samples from different parts of sub-Saharan Africa appear to be similar in population structure, though some clones have been consistently reported in only some parts of the sub-region. For example, STs 913, 925, 1737, 2160, and 3310 have so far been reported only in The Gambia, which could also be attributed to the fact that the pneumococcal population has been less studied in many parts of sub-Saharan Africa.

Little is known about molecular epidemiology of the pneumococcus in sub-Saharan Africa though majority of pneumococcal infections occur in this setting. Lack of molecular typing data of the pneumococcus in sub-Saharan Africa is mainly due to lack of facilities as well as technical expertise required for the organism. Thus capacity building in the form of training personnel on the pneumococcus, and also providing adequate research funding constitute a major part of the solution to this problem. Currently, there is an urgent need for molecular epidemiology data on the pneumococcus in sub-Saharan Africa given the expected massive pneumococcal vaccination in the region (PneumoADIP, 2007). This information is necessary to monitor evolutionary changes in the pneumococcal population following introduction of pneumococcal vaccines. Trends in evolutionary changes following pneumococcal vaccination have been documented in developing countries such as the United States (Brueggemann et al., 2007). However, the data may not be applicable to sub-Saharan Africa, as the population structure of the pneumococcus in sub-Saharan Africa appears to be quite divergent from what is known in the developed world. It is important to note that the absence of serotype 1 in the pneumococcal 7-valent vaccine (PCV7) makes the vaccine highly unsuitable for sub-Saharan Africa given the role of pneumococcal serotype 1 in epidemics in the sub-region. Additionally, the use of PCV7 in sub-Saharan Africa could select for serotype 1 with changes in its population structure, and for that matter other serotypes not included in the vaccine.

## Conclusion

Few studies have employed molecular methods to investigate the pneumococcus in sub-Saharan Africa. Consequently, little is known about molecular epidemiology of the pneumococcus in sub-Saharan Africa, though majority of pneumococcal infections occur in this region. The pneumococcal population in sub-Saharan Africa appears to be quite divergent from what is known in the developed world, and pneumococcal serotype 1 related to the ST 618 clone and clones of the ST 217 clonal complex are responsible for outbreaks in sub-Saharan Africa. There is an urgent need for molecular epidemiology data on the pneumococcus in sub-Saharan Africa to help monitor evolutionary changes in the pneumococcal population following introduction of pneumococcal vaccines in the sub region.

### Conflict of interest statement

The author declares that the research was conducted in the absence of any commercial or financial relationships that could be construed as a potential conflict of interest.
